# HunCRC: annotated pathological slides to enhance deep learning applications in colorectal cancer screening

**DOI:** 10.1038/s41597-022-01450-y

**Published:** 2022-06-28

**Authors:** Bálint Ármin Pataki, Alex Olar, Dezső Ribli, Adrián Pesti, Endre Kontsek, Benedek Gyöngyösi, Ágnes Bilecz, Tekla Kovács, Kristóf Attila Kovács, Zsófia Kramer, András Kiss, Miklós Szócska, Péter Pollner, István Csabai

**Affiliations:** 1 Department of Physics of Complex Systems, ELTE, Eötvös Loránd University, Budapest, Hungary; 2 2nd Department of Pathology, Semmelweis University, Budapest, Hungary; 3 Health Services Management Training Centre, Semmelweis University, Budapest, Hungary; 4 MTA-ELTE Statistical and Biological Physics Research Group at Dept. of Biological Physics, Eötvös Loránd University, Budapest, Hungary

**Keywords:** Cancer imaging, Pathology, Information theory and computation, Electrical and electronic engineering, Computer science

## Abstract

Histopathology is the gold standard method for staging and grading human tumors and provides critical information for the oncoteam’s decision making. Highly-trained pathologists are needed for careful microscopic analysis of the slides produced from tissue taken from biopsy. This is a time-consuming process. A reliable decision support system would assist healthcare systems that often suffer from a shortage of pathologists. Recent advances in digital pathology allow for high-resolution digitalization of pathological slides. Digital slide scanners combined with modern computer vision models, such as convolutional neural networks, can help pathologists in their everyday work, resulting in shortened diagnosis times. In this study, 200 digital whole-slide images are published which were collected via hematoxylin-eosin stained colorectal biopsy. Alongside the whole-slide images, detailed region level annotations are also provided for ten relevant pathological classes. The 200 digital slides, after pre-processing, resulted in 101,389 patches. A single patch is a 512 × 512 pixel image, covering 248 × 248 *μm*^2^ tissue area. Versions at higher resolution are available as well. Hopefully, HunCRC, this widely accessible dataset will aid future colorectal cancer computer-aided diagnosis and research.

## Abstract (summary)

**Table:** 

Measurement(s)	H&E slide staining • *ex vivo* light microscopy with immunohistochemistry and digital image analysis • Image Annotation Statement • Screening Colonoscopy
Technology Type(s)	Hematoxylin and Eosin Staining Method • bright-field microscopy • Observation • Biopsy of Colon
Factor Type(s)	screening status of colon cancer or normal tissue
Sample Characteristic - Organism	Homo sapiens
Sample Characteristic - Location	Central Hungary

## Background & Summary

Colorectal cancer (CRC) is one of the most common cancer types, especially in high-income countries, ranking fourth in mortality causing and third in incidence worldwide^1^. The advanced disease has a poor prognosis making early diagnosis crucial for long term survival.

The two main reasons for CRC screening are to reduce CRC mortality by earlier detection at a less advanced stage and to detect and remove pre-malignant lesions prior to any malignant transformation. The systematic review of Bénard *et al*.^2^ summarises the CRC screening recommendations worldwide. The recommendation of the age to start CRC screening in developed countries such as in the US and Western Europe is 50 years. The recommended frequency of re-screening depends on previous macroscopic and microscopic findings. The non-invasive first step in screening is a stool test which could be guaiac-based or immunochemical fecal occult blood testing. Following a positive result, more sensitive and sophisticated tools are required, these include; colonoscopy, colon capsule endoscopy, or computer tomographic colonoscopy.

Colonoscopy remains the gold standard for early detection during which, suspicious lesions are sampled or totally removed. The obtained tissue material is subsequently processed and histologically evaluated by pathologists. The pathological work-up aims to answer critical questions regarding the lesion of interest, these being whether the lesion is: neoplastic or non-neoplastic, benign or malignant, its degree of dysplasia, depth of invasion, histological subtype, etc. These parameters have both prognostic and therapeutic implications and are essential for successful patient management. Histological evaluation requires highly trained, specialized pathology professionals. The introduced colorectal screening programs have put an increasing workload on these professionals and there is a global pathologist workforce shortage^3^. High throughput image analysis software would help to accelerate this pathological diagnostic process.

Convolutional neural networks (CNN) are highly effective tools for many computer vision tasks. Since the early 2010s, various tasks have been undertaken, where CNNs could match or even surpass human performance in the same tasks. Most of this research was based on supervised learning. A method that requires carefully labeled datasets, on which the model could be trained.

Image-based medical diagnostics is a fruitful area of application for CNNs, however, the collection of labeled data is often difficult due to both ethical issues and the lack of available time for medical experts to label data. Therefore, publicly available, labeled medical datasets are rare.

A deep learning based polyp detection solution could predict the histological type of colonoscopy images^4^. We believe that the image-based diagnosis by an endoscopist will never be as precise as a histopathological result as the pathologist investigates samples at a much higher resolution. The sample processing routine may change in the future as more digital slides may be produced and prefiltered by a computer-aided diagnosis algorithm to facilitate a pathologists’ decision making. For this purpose, an approach to determine four stages of CRC development (normal, preneoplastic, adenoma, and cancer) was published by Sena *et al*.^5^.

The TCGA-COAD dataset^6^ contains 1419 colon whole-slide images (WSI). The majority of them, more than 950, are flash-frozen slides, and the rest are formalin-Fixed Paraffin-Embedded (FFPE). These TCGA slides originate from surgical resection and not colonoscopic examination. Also, detailed segmentation for the TCGA slides is not available, while some follow-up attempts were made to extract tumor only regions^7^ from the WSIs. The LC25000^8^, NCT-CRC-HE-100K^9^ and the Collection of textures in colorectal cancer histology^10^ datasets contain various segmented regions of colorectal cancer WSIs, but only patched datasets are available, each of them assigned to a pathological condition, the WSIs, however, are not. The DigestPath19 (https://digestpath2019.grand-challenge.org/) dataset contains patched images with pixel-level tumor segmentation masks. Outside of the colorectal datasets, the CAMELYON dataset^11^ is a good example of a high-quality, open source dataset, which published pixel-level annotated WSIs of lymph nodes, aiding metastatic breast cancer identification.

Unfortunately, publications of CNN applications in medical imaging often do not publish their data, restraining further development of such tools which could potentially help millions in the global population.

## Methods

### Scanning

200 hematoxylin-eosin (H&E) stained, FFPE colon slides were analyzed retrospectively with the permission of the National Medical Ethical Committee (32191/2019/EKU). The samples were selected from the archives of the 2nd Department of Pathology of Semmelweis University, Budapest, and were scanned with a 3DHistech Pannoramic 1000 Digital Slide Scanner. The samples were collected by colonoscopic examination. Scanning was performed with the highest available, 40x magnification, which resulted in 0.1213 *μm*/pixel resolution. Mikrozid AF alcoholic wipes and microfiber cloths were used to remove any impurities from the outer glass surface of the slides.

### Annotation

Ten relevant pathological conditions were marked by drawing (local annotations) on each WSI. For the frequency of the various annotations, see Table 1. The selected conditions are: *low-grade dysplasia*, *high-grade dysplasia*, *adenocarcinoma*, *suspicious for invasion*, *inflammation*, *resection edge*, *tumor necrosis*, *lymphovascular invasion*, *artifact* (any dirt, artificial tissue damage or other technical issues that made the diagnosis of the given area significantly harder or impossible) and *normal*. Technically, no normal tissue was annotated. Instead, all the non-annotated tissue was considered normal.

**Table 1:** Summary of the annotated data at patch level, after removing the patches that did not pass the quality filter.

label	#WSIs	#patches	%patches	#Gpixels
low-grade dysplasia	115	57397	56.61	15.0
high-grade dysplasia	35	3057	3.02	0.8
adenocarcinoma	34	4567	4.50	1.2
suspicious for invasion	13	681	0.67	0.2
inflammation	22	1026	1.01	0.3
resection edge	11	541	0.53	0.1
tumor necrosis	10	624	0.62	0.2
lymphovascular invasion	0	0	0.00	0.0
artifact	30	4169	4.11	1.1
normal	174	31323	30.89	8.2

Altogether 200 WSIs were annotated and it can be clearly seen that almost all contained some normal tissue. Note, that summing the proportions end up over 100%. This happens because some patches have overlapping, multiple annotations. Altogether 101,389 patches were stored.

In addition, four categorical characteristics were marked which globally described the WSI (global annotations). The global characteristics were the

- *Haggitt-level*^12^
- indication of the origin of the sample, options: *biopsy or polypectomy*
- *polyp type*, options: *hyperplastic, sessile serrated, traditional serrated adenoma, tubular adenoma, tubulovillous adenoma, villous adenoma, hybrid*
- *main category*, options: *normal, colorectal cancer, non neoplastic lesion, or adenoma*

All annotations were selected based on clinical relevance, general applicability, and as a result of medical consensus motivated by and following the Vienna classification^13,14^.

The desired minimal accuracy for the local annotations was 250 *μm*. In QuPath v0.1.2^15^, the software that was used for the annotation, a square grid with 250 *μm* unit length could be turned on as a scale.

All WSIs were annotated by a pathology resident physician and every single annotation was validated, and where it was needed, adjusted by a board-certified pathologist. During annotation, the original diagnosis from the retrospective database was available for the annotators.

A usual slide contains several sections of the same sample. Each time one of these sections was randomly chosen and only that section was annotated as annotating several, extremely similar samples is not an effective use of resource management.

The local annotation was performed in QuPath v0.1.2^15^, while the global annotations were collected in spreadsheets.

### Data preprocessing

The WSIs were stored in MIRAX (.mrxs) format which represents a huge data volume, in our case, the 200 WSI required 392 GB of storage space. To facilitate data handling, the raw WSIs were sliced into smaller, 512 × 512 pixel patches. Only patches that fell in the region of the section of interest, the annotated region, were kept. During the next step, for each patch, a quality filter was applied to keep or discard the given patch. The patching and the filtering were performed at three different zoom levels, for the resulting statistics, see Table 2. The 124 × 124 *μm*^2^ and the 248 × 248 *μm*^2^ patches are published as easily accessible lightweight datasets, while the 62 × 62 *μm*^2^ patch dataset can be generated using the published MIRAX digital slides and the supporting code.

**Table 2:** Summary of the patches, which passed the quality filters for three different zoom levels.

zoom ID	covered tissue per patch (*μm* × *μm*)	#patches	dataset size (GB)
0	62 × 62	1,593,113	79
1	124 × 124	402,904	24
2	248 × 248	101,389	7

For each zoom level a single patch contains 512 × 512 pixels. In the manuscript, all the results are presented using the dataset, which corresponds to the zoom ID 2.

#### Algorithm 1

Patch quality filter logic.

**Fig. 1:**
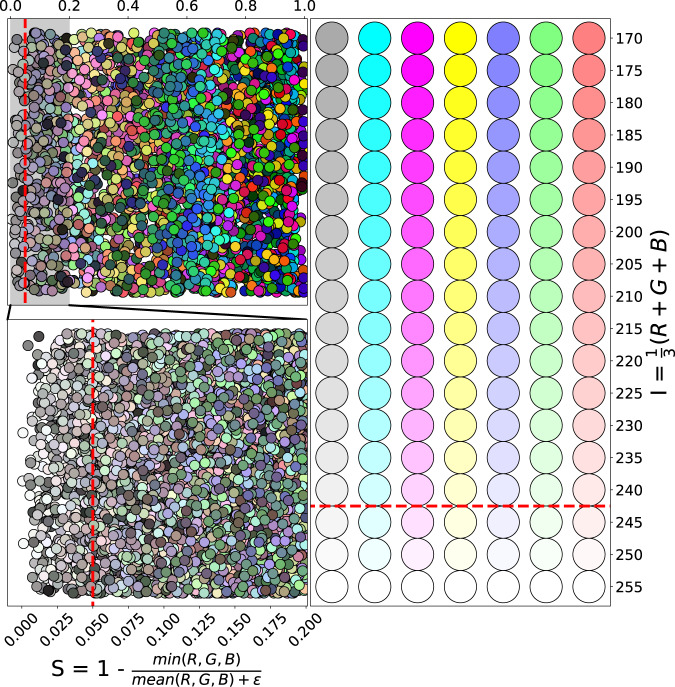
Visualization of the saturation and intensity based patch quality filter. Top Left: saturation was calculated for various RGB colors. The red dashed line shows the used threshold value 0.05, which is clearly a permissive cutoff. Bottom Left: Zoomed to the highlighted 0.0–0.2 region. *ε* = 10^−8^ is needed for numerical stability. Right: intensity plotted for a few RGB colors, the colors are considered burnout if their intensity is over 245. On the figure the red dashed line separates the burnout colors.

For the considered patches the pixel-wise intensity and saturation were calculated. Pixels were categorized as unsaturated if the saturation was lower than 0.05 and burnout if the intensity was higher than 245. For visualizations of these thresholds, see Fig. 1, where also the used intensity and saturation formulas are shown. Only patches that had less than 50% unsaturated and less than 50% burnout pixels at the same time were kept, see the pseudo-code Algorithm 1.

**Fig. 2:**
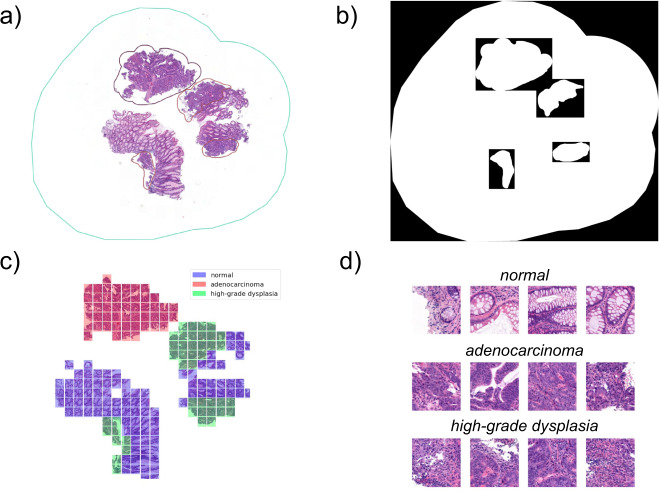
The process of creating annotations on the WSI. First, the local, free-hand annotations are marked on WSI. Then, the annotations are exported as binary masks which get processed into categorized patches with the described filtering, see Algorithm 1. (**a**) Screenshot from QuPath v0.1.2 from the viewpoint of the annotator. The surrounding cyan-blue line indicated the area that was annotated. The various smaller colored regions represent different labeled areas. A tissue area without any annotation is considered normal. (**b**) The exported binary, black&white masks for the annotations. (**c**) Visualization of patches on a WSI. Patches that did not pass the quality filter are discarded and patches that had more than half of their pixels labeled with an annotation are assigned to that category. For the shown example 186 patches were kept. (**d**) A few examples of the resulting 512 × 512 pixel size patches. Each patch covers a 248 *μm* × 248 *μm* area.

H&E staining produces a unique deep blue-purple/pink, high saturation coloring of the samples, see Fig. 2. The desired goal with this quality control was to separate the H&E stained sample regions from the background and smaller artifacts, such as dirt, scratches, and small-size non-tissue origin debris.

### Generating labels for the patches

All the drawn local annotations were extracted as black and white images, see Fig. 2, containing the bounding box area of the given annotation. The black and white pixels indicated the exact shape of the annotations. The extraction was performed with a custom extension written for QuPath v0.1.2, see the Code Availability section. A patch was assigned to one of the local categories if at least 50% of the pixels were overlapping with the drawn annotation. As a result, we acquired a patched dataset. For detailed statistics of the patches see Table 1. For each patch the coordinate of its top-left pixel was also stored, thus the reconstruction of the filtered WSI is possible.

### Baseline modeling results

To explore the possibilities and limitations of the dataset, an ImageNet pre-trained ResNet50^16^ CNN was trained and evaluated on the patched images using TensorFlow 2.0^17^. ResNet50 was selected because its popularity provides an easily accessible baseline. The evaluation was performed via 5 fold cross-validation. The 5 folds were generated WSI wise, meaning that each time patches from 40 WSIs were in the test set while patches from 160 WSIs were in the training set. It was necessary because randomly sampling from the pool of all the patches would result in overestimation of the results as the neighboring patches are often highly similar to each other, resulting in an effect known as information leakage. During both training and evaluation the patches were treated separately, no spatial information was given to the network.

The training process was performed with the stochastic gradient descent (SGD) optimizer for 15 epochs with a mini-batch size of 16. The learning rate was set to 10^−3^ for the first 10 epochs and was then dropped to 10^−4^ for the remaining 5 epochs. During training, the images were randomly flipped and transposed as augmentations (one of the 8 possible configurations was selected randomly) because orientation is a natural symmetry for these images. Binary cross-entropy loss was used to train the neural network for the multi-label classification task because patches could belong to several categories at the same time.

**Fig. 3:**
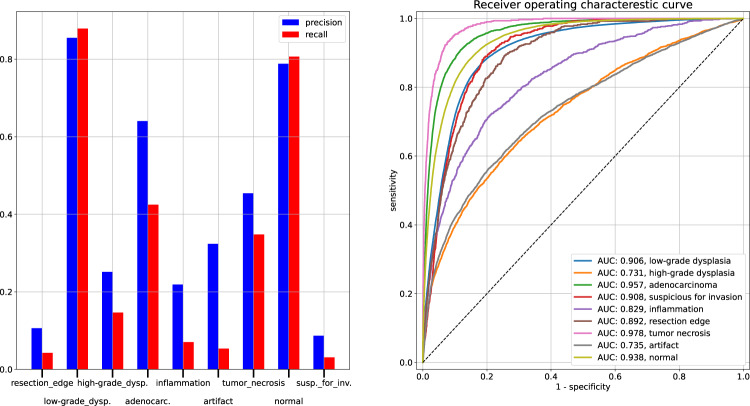
Predictions generated by a ResNet50 CNN with 5-fold cross-validation, calculated from patch-level data. All patches were handled independently. Left: precision and recall score for various local annotation categories. The outputs of the final sigmoid layer, the probability predictions were converted to binary predictions with a 50% threshold. Right: receiver operating characteristic (ROC) curves for the same categories.

The model resulted in a high area under the receiver operating characteristic curve (AUC) score, ranging from 0.73 to 0.98 for the differing medical classes, see Fig. 3. For the *normal* and *low-grade dysplasia* patches, the model reached around 0.8 precision and recall and performed more poorly for the other, less frequent labels.

Limitations of the baseline model are clearly visible: while it works well for classes that have a large amount of data available (*normal* and *low-grade dysplasia*), it performs worse for conditions which are rarer. While our dataset is large in data volume, it is still limited to only 200 WSIs, meaning that the possible variations in sample preparation and the anatomical variations are not fully represented.

More sophisticated modeling approaches might gain a significant performance boost by using spatial information, which is an ongoing research task for this dataset along with the exploration of the possibilities of a decision support system based on the collected data. The sole purpose of providing the patch-level ResNet50 results is to set up a default baseline for the dataset.

## Data Records

The HunCRC patched dataset is saved and shared in standard JPEG format as a Figshare dataset of this paper^18^. In the repository, all the patches can be found that passed the filtering process in JPEG format for the zoom ID 1 and 2. Also, standard CSV files are available that contain all the accompanying meta-data, as well as the matched local categories, the position of the patch within the WSI and the global annotations. An additional CSV file of metadata contains the age, sex and ICD-10 health status code of the patients. Patients are referenced with an anonymous numeric identifier in the range of 001 and 200.

The unprocessed raw scans in MIRAX (.mrxs) format alongside the unprocessed QuPath v0.1.2 annotations, and the exported annotations as pixel-masks, are available through The Cancer Imaging Archive (TCIA)^19^.

## Technical Validation

The used pathological slides went through multiple round quality controls. First, they were used for diagnostic purposes without digitization which proves the quality of the sections. After digital scanning, the slides were annotated by pathology resident physicians. During the annotation, the blurred, low-quality WSIs were identified and sent back for re-scanning. For the last step, all the annotations on the high quality WSIs were validated and adjusted, when needed, by a board certified pathologist.

## Usage Notes

The provided patches are saved in standard JPEG format images and the metadata is provided as a CSV data file. No special software is needed to handle these data types.

In the metadata, each patch is represented by a row, where the filename of the JPEG patch is linked to the annotation labels and to the top-left coordinates of the patch. Using this information, re-building or re-scaling the annotated and filtered part of the WSI is achievable from the provided patches. For each WSI, an overview image is provided as a screenshot with annotation and as a thumbnail image without annotation.

The raw scans are shared in MIRAX (.mrxs) format, which can be accessed by any standard digital pathological software. The unprocessed pixel-level annotations require QuPath v0.1.2, however, for machine processing, these annotations are also provided as exported PNG binary masks with filenames containing the labels and position information.

The presented HunCRC data is available under CC BY 4.0 licence.

## Data Availability

The code that exports the QuPath v0.1.2 manual annotations is accessible at https://github.com/qbeer/qupath-binarymask-extension. The required system memory is at least 8GB. The annotations are saved as binary masks for each corresponding category and at a predefined sub-sampling rate based on spatial size. All these metadata are stored in the filename with the location of the top-left pixel in the original whole slide at the highest resolution. The patch generating and CNN modeling code is available at http://github.com/patbaa/crc_data_paper. The code processes the whole slides in a sequential manner, patch-by-patch, and does the above-described filtering steps (Algorithm 1) while it also assigns all the corresponding labels to patches. The code for the baseline CNN training also can be found in the same repository.
